# A novel T cell-redirecting anti-GPRC5D × CD3 bispecific antibody with potent antitumor activity in multiple myeloma preclinical models

**DOI:** 10.1038/s41598-024-55143-0

**Published:** 2024-03-01

**Authors:** Urara Tomita, Yoko Ishimoto, Masaki Ri, Yumi Kawase, Yoshiyuki Hizukuri, Chikako Maru, Kayoko Nanai, Ryuichi Nakamura, Makiko Nakayama, Keiko Oguchi-Oshima, Hiroyuki Sumi, Toshiaki Ohtsuka, Shinsuke Iida, Toshinori Agatsuma

**Affiliations:** 1 Daiichi Sankyo Co., Ltd., Tokyo, Japan; 2 Department of Hematology and Oncology, Nagoya City University Graduate School of Medical Sciences, Nagoya, Aichi Japan; 3 Daiichi Sankyo RD Novare Co., Ltd., Tokyo, Japan

**Keywords:** Myeloma, Targeted therapies, Cancer immunotherapy, Antibody therapy

## Abstract

G-protein-coupled receptor class 5 member D (GPRC5D) is detected in malignant plasma cells in approximately 90% of patients diagnosed with multiple myeloma (MM). Here, we constructed BsAb5003, a novel humanized bispecific monoclonal antibody targeting CD3 and GPRC5D, and evaluated its therapeutic impact on MM. BsAb5003 induced specific cytotoxicity of GPRC5D-positive MM cells with concomitant T cell activation and cytokine release. The efficacy of BsAb5003 was associated with GPRC5D expression levels in MM cell lines. Flow cytometry analysis of bone marrow mononuclear cells (BMMNCs) from 49 MM patients revealed that GPRC5D was expressed in a wide population of MM patients, including heavily treated and high-risk patients. In ex vivo assays using BMMNCs, BsAb5003 induced potent efficacy against CD138 + MM cells in both newly diagnosed and relapsed/refractory patient samples in a GPRC5D expression-dependent manner. BsAb5003 significantly enhanced T cell activation and cytokine production in combination with immunomodulatory drugs (IMiDs) against MM cell lines. BsAb5003 also demonstrated significant inhibition of in vivo tumor growth by recruiting T cells. Taken together, these results suggest that T cell-redirecting bispecific antibody targeting GPRC5D as monotherapy and combination therapy with IMiDs could be a highly potent and effective treatment approach for a wide population of MM patients.

## Introduction

Multiple myeloma (MM) is highly complex at diagnosis, at relapse, and during the disease course due to genomic events and clonal evolution with numerous mechanisms of resistance^1^. Despite the increasing availability of effective therapies for patients with MM that provide longer disease-free periods, the vast majority still suffer multiple relapses and remain incurable. Approximately 10%–20% of patients with high-risk factors show a poorer outcome than expected, with death occurring within 3 years of diagnosis^2,3^. There is thus an urgent need for new therapies, in particular therapies that address novel targets and/or with new mechanisms of action, to overcome the unavoidable resistance to current agents^4^.

G-protein-coupled receptor class 5 member D (GPRC5D) is an orphan receptor and a seven-trans membrane protein that is predominantly expressed in cells with a plasma cell phenotype, including the majority of malignant plasma cells from patients with MM, which are defined as CD138 + MM cells^5,6^. It has been reported that approximately 90% of patients with MM exhibit the expression of GPRC5D in MM cells^7,8^, but shows only low/limited expression in normal cells such as those in hard keratinized structures including the hair shaft, nail, and central region of the tongue^9^. The selective expression of GPRC5D could thus be an ideal target for effector cell-mediated therapy such as T cell-redirecting bispecific antibodies and CAR-T cells^10–12^. Indeed, recently Talquetamab, a T cell-redirecting bispecific antibody targeting GPRC5D, have received accelerated FDA approval for the treatment of relapsed/refractory (RR) MM^13^, and several other therapeutics targeting GPRC5D have also achieved encouraging results in clinical studies^14,15^. However, there are still several challenges to overcome and opportunities to exploit, such as mechanisms of resistance^16^, potential rational combinations^17^, and mitigation of toxicities including cytokine release syndrome^18^.

We constructed BsAb5003, a novel humanized bispecific monoclonal antibody that binds to CD3 on T cells and GPRC5D on plasma cells. BsAb5003 comprises three distinct components: a humanized anti-CD3 single-chain variable fragment (scFv) domain, derived from a novel CD3 antibody with relatively low affinity for CD3 to optimize both efficacy and safety^19^; a humanized anti-GPRC5D Fab domain, which binds to N-terminal region of GPRC5D and exhibits a strong target binding signal, aligning with the understanding that target tumor-associated antigen (TAA) affinity correlates with target-dependent killing activity by T cell engagers, even for low target density^20,21^; and a hetero-Fc domain with effectorless mutations based on Azymetric™ and EFECT™ platforms^22,23^ designed to prevent Fcγ receptors and complement bindings, and to mitigate the risk of TAA-independent cytokine production. Overall, these components were careful combined in BsAb5003 to maximizes its efficacy and safety.

Here we evaluated the preclinical activity of BsAb5003 in MM cell lines and in MM patient samples with high to low GPRC5D expression. Moreover, we investigated the effect of combining BsAb5003 with immunomodulatory drugs (IMiDs) or proteasome inhibitors (PIs) to provide a preclinical rationale for its potential use with those current backbone therapies for MM^24^.

## Results

### Affinity and in vitro anti-tumor efficacy of BsAb5003

**Figure 1:**
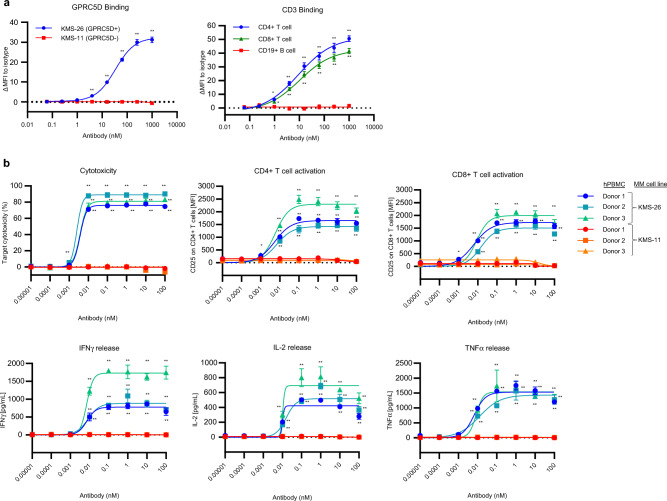
Binding profile and in vitro efficacy of BsAb5003. (**a**) The binding profile of BsAb5003 on target cells was analyzed using flow cytometry. The binding activity of various concentrations of BsAb5003 on MM cell lines, KMS-11 and KMS-26 (left), or CD4 + T, CD8 + T, and CD19 + B cells among hPBMCs (right) were plotted as change of median fluorescence intensity (ΔMFI). ΔMFI was calculated by subtracting MFI of isotype antibody-treated sample from MFI of BsAb5003-treated sample. Statistical significance (compared to 0.061 pM group) was determined using a one-tailed Williams test (*P < 0.025, **P < 0.005). (**b**) GPRC5D-positive KMS-26 cells and GPRC5D-negative KMS-11 cells were cocultured with hPBMCs from three healthy volunteers with serial dilution of BsAb5003. Culture supernatants were collected and IFNγ, IL-2, and TNFα were measured using a Luminex system on day 1(bottom three figures). Cytotoxicity, CD4 + T cell activation and CD8 + T cell activation were measured by flow cytometry on day 3(top three figures). The assay was conducted in triplicate and the data represent the mean ± standard error. Statistical significance of BsAb5003 treatment was determined using a one-tailed Williams test (*P < 0.025, **P < 0.005).

We developed BsAb5003, a novel humanized Fab-scFv anti-GPRC5D × CD3 bispecific antibody. To confirm the binding affinity to GPRC5D, we performed FCM analysis using KMS-11 and KMS-26 cells as GPRC5D-negative and positive cells, respectively, in accordance with the expression data of the Cancer Dependency Map^25^. As expected, BsAb5003 bound to KMS-26 but not to KMS-11 (Fig. 1a left). We also confirmed the binding of BsAb5003 to the N-terminal peptide of GPRC5D, but not of GPRC5A, a homolog of GPRC5D (Supplemental Fig. 1), suggesting binding specificity. Binding studies by FCM also showed that BsAb5003 only bound to CD4 + T and CD8 + T cells equally due to the presence of CD3 on these cells, but not to CD19 + B cells in hPBMCs as expected (Fig. 1a right).

To evaluate in vitro target-dependent cell cytotoxicity accompanied by the T cell activation of BsAb5003, we co-cultured GPRC5D-positive KMS-26 cells or GPRC5D-negative KMS-11 cells with hPBMCs in the presence or absence of BsAb5003. BsAb5003 induced target cell cytotoxicity, both CD4 + and CD8 + T cell activation, and cytokine release in a dose-dependent manner in GPRC5D-positive KMS-26 cells (Fig. 1b hPBMC Donor1 ~ 3 with KMS-26), but not in GPRC5D-negative KMS-11 cells (Fig. 1b hPBMC Donor1 ~ 3 with KMS-11). These results indicated that BsAb5003 induces a T cell-mediated anti-tumor effect in a GPRC5D-specific manner.

### Correlation between the GPRC5D expression level and the activity of BsAb5003

**Figure 2:**
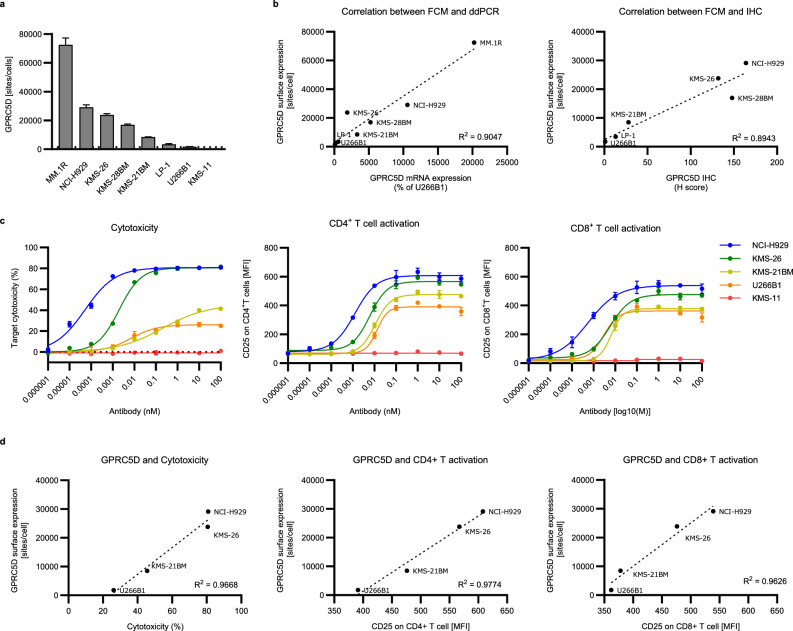
Correlation between expression of GPRC5D and efficacy of BsAb5003 in several MM cell lines. (**a**) The expression level of GPRC5D on eight MM cell lines (MM1R, NCI-H929, KMS-26, KMS-28BM, KMS-21 BM, LP-1, U266B1, and KMS-11) was measured by flow cytometry. GPRC5D antibody binding sites/cells were determined using QIFIKIT. Samples were prepared in triplicate and the data represent the mean ± standard deviation. Dotted line represents the lower limit of detection of antibody binding capacity (1500 sites/cell) and U266B1 had a value of 1774 sites/cell, which was just above the detection limit. (**b**) Correlation between GPRC5D expression level detected by FCM and relative mRNA expression from ddPCR or IHC score. Seven MM cell lines expressing GPRC5D above detectable limit (1500 sites/cell) are plotted. (**c**) Several MM cell lines were cocultured with hPBMCs at an ET ratio of 5:1 for 3 days, after which target cytotoxicity, CD4 + T cell activation, and CD8 + T cell activation were measured by flow cytometry. The assay was conducted in triplicate and the data represent the mean ± standard error. (**d**) Correlation diagram between target expression and estimated maximum efficacy of BsAb5003-induced cytotoxicity.

We next explored the relationship between the expression level of GPRC5D and the efficacy of BsAb5003, such as MM cell cytotoxicity and T cell activation. The wide variety of GPRC5D expression levels on several MM cell lines was evaluated by following three approaches: 1) FCM using indirect immunofluorescence assay to determine cell surface GPRC5D protein expression quantitatively, 2) droplet digital PCR (ddPCR) to determine GPRC5D mRNA expression level, and 3) IHC to determine the positivity of GPRC5D among cells (Fig. 2a, Supplemental Fig. 2). We found that the GPRC5D expression level on the cell surface quantified by FCM was associated with the GPRC5D mRNA expression level and the H score of GPRC5D determined by IHC (Fig. 2b). GPRC5D-specific MM cell cytotoxicity, and CD4 + T and CD8 + T cell activation were observed in a dose-dependent manner in cell lines with different GPRC5D expression levels (Fig. 2c). Notably, GPRC5D cell surface expression levels had significant positive correlations with E_max_ in MM cell cytotoxicity, and with CD4 + T and CD8 + T cell activation (Fig. 2d). These results indicated that GPRC5D expression level appears to be one of the key factors to predict of BsAb5003 activity.

### Anti-tumor efficacy in patient-derived BMMNCs by ex vivo assay

**Figure 3:**
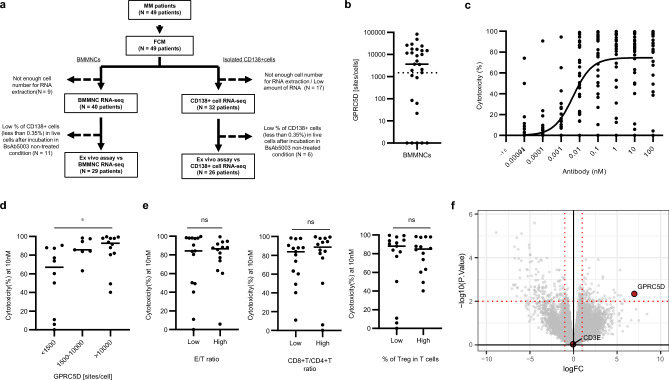
Ex vivo efficacy of BsAb5003 against patient-derived BMMNCs. (**a**) Flow of MM patient sample studies. Cytotoxicity of BMMNCs was analyzed for samples containing more than 0.35% live CD138 + cells after incubation under BsAb5003-untreated conditions (29 patients). RNA-seq data of BMMNCs from the corresponding 29 patients were used for analysis. RNA-seq data of CD138 + cells isolated from BMMNCs were also obtained, and those of 26 patients were used for analysis. (**b**) Surface GPRC5D expression level on CD138 + MM cells evaluated by flow cytometry using QIFIKIT was plotted. (**c**) MM patient-derived BMMNCs were incubated for 2−4 days with serial dilution of BsAb5003. Individual values of MM cell cytotoxicity of all 29 MM patients’ samples in each assay were plotted. A non-linear sigmoid curve for all samples (n = 29) is also indicated in the plot. (**d**) Cytotoxicity induced by 10 nM BsAb5003 treatment was compared among three groups with different GPRC5D expression (< 1500 sites/cell, 1500−10 000 sites/cell, and > 10 000 sites/cell). Statistical significance among the three groups was determined using a Steel–Dwass test (*P < 0.05). (**e**) Median E/T ratio, CD8 + T/CD4 + T ratio and Treg proportion among T cells were each used to divide the subjects into two groups, which were then compared in terms of the cytotoxicity induced by 10 nM BsAb5003 treatment. Statistical significance was determined by a two tailed Wilcoxon test (ns: *P* > 0.05). (**f**) Linear regression showing genes whose expression changed in relation to drug effect. The x-axis shows the change in gene expression for a one-unit change in MM cell cytotoxicity as a fold change. The y-axis shows the log10 of the p value as the significance of the change of gene expression. The vertical and the horizontal red dotted lines indicate a twofold change and a p value of 0.01, respectively.

We investigated the impact of tumor and immune cell context by ex vivo assay using patient-derived BMMNCs, which is a promising method to pre-clinically evaluate the effects of T cell-redirecting bispecific antibodies^26,27^. The overall study design and patient clinical characteristics are shown in Fig. 3a and Table 1.

**Table 1:** The clinical background of patient specimens.

Characteristic	All	BMMNC RNA-seq	CD138 + cell RNA-seq
	(n = 49)	(n = 29)	(n = 26)
Median age, year (range)	71 (37–86)	73 (49–86)	72 (49–86)
Age > 75 years	18 (36.7)	12 (41.4)	10 (38.5)
Age ≦75 years	31 (63.3)	27 (93.1)	16 (61.5)
Male	25 (51.0)	14 (48.3)	12 (46.2)
Female	24 (49.0)	15 (51.7)	14 (53.8)
ISS stage			
I	9 (18.4)	4 (13.8)	3 (11.5)
II	27 (55.1)	18 (62.1)	16 (61.5)
III	13 (26.5)	7 (24.1)	7 (26.9)
High-risk cytogenetics*	17 (34.7)	13 (44.8)	13 (50.0)
Median no. of prior anti-MM regimens, n (range)	1.00 (0–8)	1.00 (0–7)	1.50 (0–7)
Prior anti-MM regimens			
ND	17 (34.7)	11 (37.9)	10 (38.5)
1	8 (16.3)	5 (17.2)	3 (11.5)
2	6 (12.2)	5 (17.2)	4 (15.4)
3 +	18 (36.7)	8 (27.6)	9 (34.6)
Prior therapy			
PIs exposed	31 (63.3)	17 (58.6)	15 (57.7)
IMiDs exposed	23 (46.9)	12 (41.4)	12 (46.2)
Anti-CD38 mAb exposed	21 (42.9)	12 (41.4)	12 (46.2)
BCMA-CART exposed	3 (6.1)	3 (10.3)	3 (11.5)
Bortezomib exposed	31 (63.3)	17 (58.6)	15 (57.7)
Lenalidmide exposed	23 (46.9)	12 (41.4)	12 (46.2)
Pomalidmide exposed	11 (22.4)	7 (24.1)	6 (23.1)
Daratuzumab exposed	20 (40.8)	11 (37.9)	11 (42.3)
Double class^†^	22 (44.9)	11 (37.9)	11 (42.3)
Triple class^‡^	18 (36.7)	9 (31.0)	9 (34.6)
Prior autologous SCT	15 (30.6)	7 (24.1)	7 (26.9)
Refractory status			
Double class refractory	15 (30.6)	8 (27.6)	8 (30.8)
Triple class refractory	13 (26.5)	7 (24.1)	7 (26.9)

Data are presented as unweighted number (percentage) of patients unless otherwise indicated.

*More than one chromosomal abnormalities t(4;14), t(14;16), t(14,20), del 17p, 1q gains.

^†^Double class: PIs plus IMiDs exposed.

^‡^Triple class: PIs, IMiDs and anti-CD38 Ab exposed.

By FCM analysis, we observed a wide variety of surface GPRC5D expression levels on patient-derived BMMNCs (Fig. 3b, Supplemental Fig. 3a). BsAb5003 showed cytotoxicity to primary MM cells in a concentration-dependent manner to reach a plateau at a concentration as high as 10 nM (Fig. 3c). BsAb5003 depleted more than 50% of CD138 in samples that showed expression below the detection limit (< 1500 sites/cell) in the flow analysis, indicating a higher sensitivity in killing (Fig. 3d). Therefore, we could not define the lower limit of surface GPRC5D expression for the selection of effective sample by flow analysis (Supplemental Fig. 3a). Meanwhile, correlation between surface GPRC5D expression and GPRC5D mRNA expression level in patient derived CD138 + cell was confirmed (Supplemental Fig. 3b). When the samples were divided into three groups by GPRC5D expression level in BMMNCs, the high-GPRC5D-expression group showed a significantly higher response than the low-GPRC5D-expression group (Fig. 3d, median value of > 10 000 sites/cell; 92.8%, median value of < 1500 sites/cell; 67.0%, *P* = 0.022). To investigate the potential impacts of effector-to-T cell (E/T) ratio, CD8 + T cell-to-CD4 + T cell (CD8 + T/CD4 + T) ratio, and proportion of Tregs among all T cells, samples were divided into two groups based on the median values of each of these parameters. Our findings indicate that none of these parameters had an impact on the cytotoxicity of BsAb5003 to primary MM cells (Fig. 3e).

As shown in the volcano plot (Fig. 3f), the mRNA expression level of GPRC5D was significantly correlated with the cytotoxicity to primary MM cells, but this correlation was not found for mRNA level of CD3e, which is the other target molecule of BsAb5003. Linear regression analysis of the relationship between drug efficacy and the expression level of each gene showed that GPRC5D was strongly related to drug efficacy (Supplemental Fig. 4a). On the other hand, there was no relationship between CD3e and drug efficacy (Supplemental Fig. 4b).

### Identification of the potential target patient subset for BsAb5003

**Figure 4:**
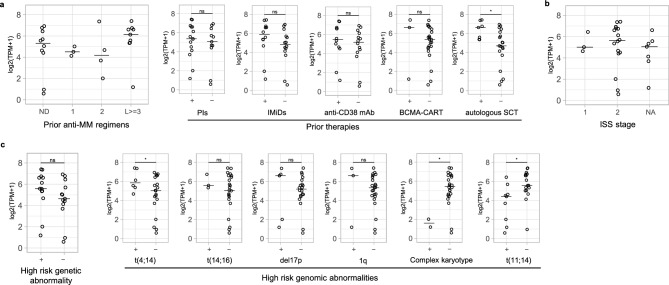
Identification of the potential target patient subset for BsAb5003. GPRC5D mRNA expression based on (**a**) treatment backgrounds, (**b**) ISS, and (**c**) genetic abnormalities in CD138 + cells sorted from BMMNCs derived from 26 patients. The samples which have been treated with a certain SoC or the presence or absence of high-risk genetic abnormality are classified in “+”, and that which have not been are in “−” (**a**,**c**). The samples which were newly diagnosed and have been treated with no treatment, that which have been with 1 regimen, that with 2 regimens, or that with 3 or more regimens are classified as “ND”, “1”, “2”, “L >  = 3”, respectively (**b**). The horizontal bar indicates median value. Statistical significance was determined by a two tailed t-test (*P < 0.05).

To identify the potential target patient subset for BsAb5003, we further analyzed the association of the expression level of GPRC5D mRNA and patient clinical characteristics, such as the line of therapy and prior therapy history. GPRC5D expression was found in various patients, ranging from those newly diagnosed (ND) to RR patients. Notably, equivalent expression levels of GPRC5D were confirmed in the patient samples with standard-of-care (SoC) drug treatments such as with proteasome inhibitors (PIs), immunomodulatory drugs (IMiDs), anti-CD38 antibody, B-cell maturation antigen (BCMA) targeting therapy. GPRC5D expression was elevated in post-autologous stem cell transplantation (ASCT) (Fig. 4a, post ASCT, *P* = 0.003), which suggests that BsAb5003 is efficacious not only in early-line but also in late-line MM patients. We also focused on several prognostic and high-risk factors to determine the expression of GPRC5D in MM patients with high unmet medical needs. GPRC5D expression was also the same among the different Internal Staging System (ISS) stages (Fig. 4b). Interestingly, GPRC5D expression was significantly higher in the patients with t(4,14), which is classified as a high-risk cytogenetic abnormality (Fig. 4c). Meanwhile, GPRC5D expression was lower in the patients with t(11,14), which is classified as a standard-risk abnormality.

Taken together, these results indicated that BsAb5003 has the potential to deplete malignant cells in ND-/ RR-MM patients, including SoC-resistant, and post-BCMA patients, and those with high-risk cytogenetics.

### Effect of combining BsAb5003 with immunomodulatory drugs and proteasome inhibitors

**Figure 5:**
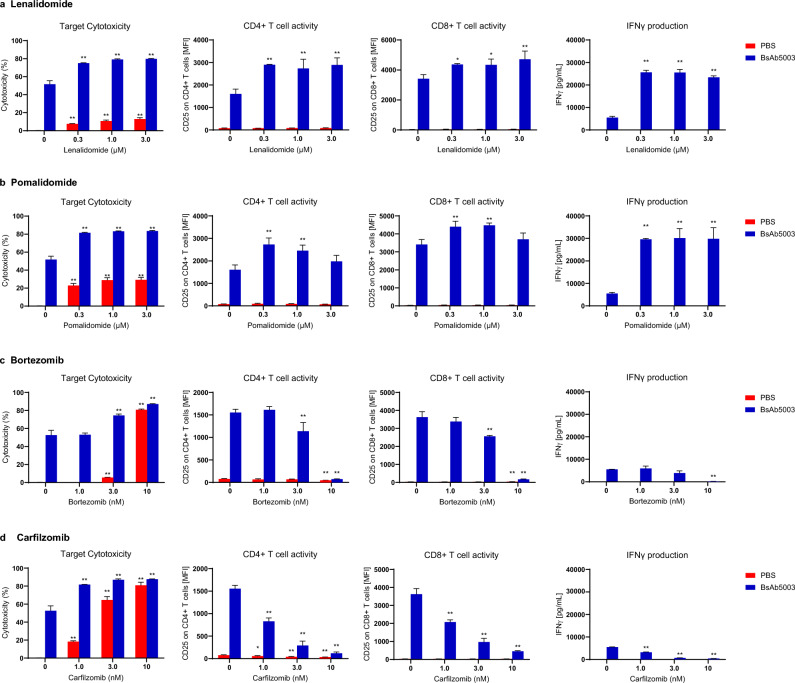
In vitro effects of combining BsAb5003 with several MM treatment drugs. OPM-2 cells were co-cultured with hPBMCs with (blue bar) or without (red bar) 5 pM of BsAb5003 in combination with (**a**) lenalidomide, (**b**) pomalidomide, (**c**) bortezomib, or (**d**) carfilzomib. On the first day of incubation, culture supernatants were collected for cytokine measurement using the Luminex system. After 3 days of incubation, cells were collected for cytotoxicity, CD4 + T cell and CD8 + T cell activation measurements by flow cytometry. The assay was conducted in triplicate or duplicate and the data represent the mean ± standard error. The statistical significance between the control sample and the samples treated with lenalidomide, pomalidomide, bortezomib, or carfilzomib with PBS or BsAb5003, respectively, was assessed using the parametric Dunnett’s test (*P < 0.05, **P < 0.01).

It has been identified that celebron (CRBN), a substrate receptor of the cullin-RING ubiquitin ligase complex, is a primary target of IMiDs and mediates various pharmacological activities including stimulation of effective T cells^28,29^. Consistent with this mechanism, it has been reported that lenalidomide or pomalidomide enhanced the lysis of MM cells mediated by the serum half-life-extended anti-BCMA BiTE^30^. To assess the potential benefit for other T cell-redirecting therapies, we evaluated the effect of combining BsAb5003 with lenalidomide or pomalidomide in a 3-day co-culture assay with human PBMCs and MM cell lines; OPM-2, KMS-26 (GPRC5D-positive) or KMS-11(GPRC5D-negative)^25^. Both OPM-2 and KMS-11 have been shown to be sensitive to IMiDs in previous experiments^31,32^. Therefore, comparing the efficacy between these cell lines allows us to anticipate the potential combination effect of BsAb5003 with IMiDs. Under our experimental conditions, the effect of lenalidomide or pomalidomide alone against OPM-2, KMS-26 and KMS-11 cells was marginal (Fig. 5a,b, Supplemental Fig. 5a,b). Under suboptimal test conditions comprising a lower BsAb5003 concentration of 5 pM, BsAb5003 alone induced approximately 50% cytotoxicity against GPRC5D-positive OPM2 and KMS-26 cells but did not induce cytotoxicity against GPRC5D-negative KMS-11 cells. Compared with BsAb5003 alone, the combination treatment with lenalidomide in GPRC5D-positive OPM-2 and KMS-26 cells induced higher tumor cell cytotoxicity than expected independent effect determined by Bliss independent model (Supplemental Fig. 6a; dotted lines), indicating synergism between BsAb5003 and lenalidomide. In addition to the synergistic cytotoxicity enhancement, increased both CD4 + and CD8 + T cell activation and IFNγ production were also observed (Fig. 5a, Supplemental Fig. 5a). Similar synergistic cytotoxicity enhancement with T cell activation and cytokine production were observed upon combination treatment with BsAb5003 and pomalidomide as well (Fig. 5b, Supplemental Figs. 5b, 6b). These results suggested the therapeutic potential of BsAb5003 in combination with IMiDs.

We also evaluated the effect of combining BsAb5003 with PIs, such as bortezomib or carfilzomib^33,34^. PIs selectively inhibit proliferation and induce apoptosis in MM cell lines and patient tumor cells^35^. Our study demonstrated that PI monotherapy induced strong dose-dependent cytotoxicity against MM cell lines regardless of GPRC5D expression (Fig. 5c,d, Supplemental Fig. 5c,d). The combination of BsAb5003 with PIs moderately enhanced BsAb5003-mediated cytotoxicity against MM cell lines at lower PI concentrations. However, treatment in combination with PIs at higher PI doses displayed antagonistic effect in cytotoxicity (Supplemental Fig. 6c, d) and strongly attenuated T cell activation (Fig. 5c,d, Supplemental Fig. 5c,d), which may limit the efficacy of BsAb5003. These results suggest the importance of selecting appropriate dose ranges of PIs in combination with T cell-redirecting bispecific antibodies to obtain the best potency with less toxicity.

**Figure 6:**
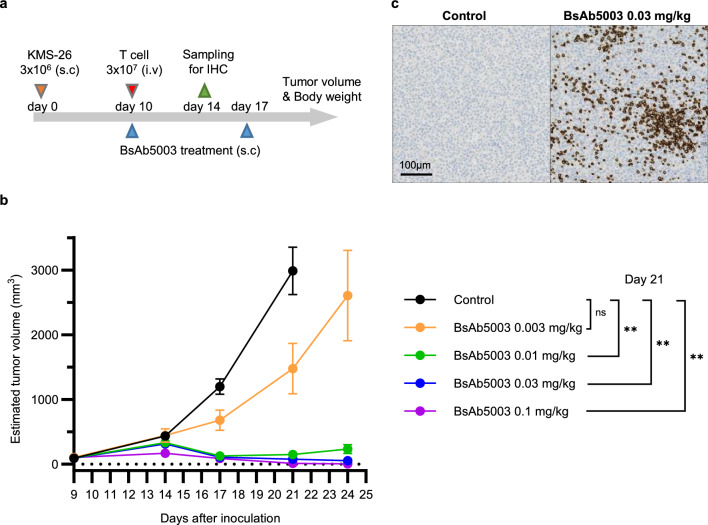
Antitumor activity of BsAb5003 against KMS-26 tumors in NSG mice inoculated with human T cells. (**a**) Each mouse received the SC injection of 3 × 10^6^ KMS-26 cells into the left flank on day 0. Ten days later, mice were injected IV with 1 × 10^7^ human T cells, followed by IV administration of PBS as a control or BsAb5003 antibody at 0.003 mg/kg, 0.01 mg/kg, 0.03 mg/kg or 0.1 mg/kg on days 10 and 17. (**b**) Tumor size was measured twice a week. Each plot indicates mean ± standard error of estimated tumor volume (n = 6). Statistical significance between the control group and treatment groups with 0.01 mg/kg, 0.03 mg/kg, and 0.1 mg/kg BsAb5003 at day 21 was assessed using parametric Dunnett's test (**P < 0.01). (**c**) For IHC staining, mice were sacrificed by carbon dioxide inhalation on day 24 and the tumors were obtained. Representative images of CD3 IHC in control mouse (left) and mouse treated with 0.03 mg/kg BsAb5003 are shown.

### Efficacy of BsAb5003 in human T cell-transferred xenograft mouse model

To evaluate the in vivo anti-tumor activity of BsAb5003, we used mouse xenograft tumor of GPRC5D-expressing KMS-26 cells in NSG mice transferred with human T cells (Fig. 6a, Supplemental Fig. 7). The results showed significant inhibition of tumor growth with the administration of BsAb5003 (Fig. 6b) and confirmed the correlation between BsAb5003 treatment dose (0, 0.003, 0.01, 0.03, 0.1 mg/kg) and tumor volume on Day 21 (Spearman’s rank correlation coefficient; − 0.9141, *P* < 0.0001). Strong anti-tumor efficacy with complete remission (estimated tumor volume of 0 mm^3^) was observed in 5/6 mice at 0.1 mg/kg on day 24. In addition, 14 days after BsAb5003 treatment, intensive CD3-positive cell infiltration was observed in the tumor area (Fig. 6c). These results indicated that BsAb5003 redirects T cells to the GPRC5D-expressing tumor in vivo and induces significant tumor growth inhibition and regression in human multiple myeloma.

## Discussion

The results of in vitro and in vivo assays in this study confirmed that our novel bispecific antibody, BsAb5003, has an adequate profile as a T cell-redirecting bispecific antibody. Several studies evaluating different T cell-redirecting bispecific antibodies reported that they activate both CD4 + and CD8 + T cells, which contribute to target cytotoxicity^36,37^, and their activity correlates with tumor antigen expression^38–40^. Consistent with these studies, we demonstrated that response induced by BsAb5003 treatment also strongly correlated with GPRC5D expression in an in vitro assay using various MM cell lines. In addition, in line with the feature of the T cell-redirecting bispecific antibodies^41,42^, BsAb5003 also induce cytotoxicity against target cells with minimal detection limit level of GPRC5D, suggesting its potential to deplete MM cells expressing very low levels of GPRC5D. In contrast, when GPRC5D expression is absent, no cell cytotoxicity or T cell activation is observed due to the incorporation of effectorless mutations in BsAb5003, which is designed to avoid inducing antibody-dependent cellular cytotoxicity and complement-dependent cytotoxicity by exhibiting minimal binding to complement or Fc receptors^43,44^. These findings suggest that BsAb5003 has minimal off-target effects.

BsAb5003 showed strong efficacy in MM patient-derived BM samples, which are a mixture of several types of cells including MM cells, effector cells, and immunosuppressive cells, while also showing intra-individual differences^45^. Linear regression analysis using both efficacy data of BsAb5003 and RNA-seq data demonstrated that GPRC5D expression is the most effective predictor of drug efficacy. The reduction in plasma cells to 5% or less on bone marrow aspirates is one of the criteria of complete response (CR) in the International Response Criteria for multiple myeloma^46^. In our study, we found that 42% of high-GPRC5D-expression group (> 10 000 site/cell), 29% in medium-GPRC5D expression group (1500−10 000 sites/cell), and 0% in low-GPRC5D expression group (< 1500 sites/cell), respectively, showed residual CD138 levels below 5% after treatment with BsAb5003 at 10 nM (Fig. 3d). We also found that 36% of ND patient samples and 44% of RR patients samples belonged to high-GPRC5D expression group, indicating that prior regimens do not reduce the expression of GPRC5D (Data not shown). These results suggest that both ND and RR patients with high-GPRC5D-expression can expect strong therapeutic effect equivalent to CR with higher probability. Interestingly, our retrospective analysis revealed that a high-risk chromosome abnormality, t(4,14), provokes an increase of GPRC5D expression, which is consistent with a previous study on 48 MM patients in Vienna^5^. This indicates that GPRC5D targeted T cell-redirecting bispecific antibodies are applicable or even preferable for use in patients with high-risk chromosome abnormalities with high unmet medical needs. Meanwhile, further research is needed to understand the role of these chromosome abnormalities in GPRC5D expression.

We further analyzed the impact of immune status on drug efficacy. Here, we used xCELL to estimate the proportion of each immune cell type in BMMNCs derived from each MM patient. xCELL is a gene-signature-based cell type enrichment tool, in which 64 immune and stroma cell types including composite scores derived from several cell type enrichment scores were obtained from a bulk RNA-seq dataset^47^. Immune score is one of a composite score based on the enrichment scores of several cell types, such as B cells, CD4 + T cells, CD8 + T cells, dendritic cells, eosinophils, macrophages, monocytes, mast cells, neutrophils, and natural killer cells, and it is used as an index representing immune status. In contrast to a previous study^48^, our results revealed that immune status is not correlated with drug efficacy (Supplemental Fig. 4a). On the other hand, we also noticed that expression and efficacy did not correlate well in a few samples. Therefore, we focused on the sample with the lowest GPRC5D among those with more than 50% of MM cell cytotoxicity as “GPRC5D low, Efficacy high”, and the sample with the highest GPRC5D among those with less than 50% of MM cell cytotoxicity as “GPRC5D high, Efficacy low”. Interestingly, the “GPRC5D high, Efficacy low” sample showed a low immune score, and the “GPRC5D low, Efficacy high” sample showed a high immune score (Supplemental Fig. 4c), indicating that immune cells are contributing to the anti-tumor activity induced by BsAb5003. Although it is necessary to gain more insights into the relationship among GPRC5D expression, cytotoxicity, and immune cell profiles to obtain more robust results, these data from outliers revealed the involvement of immune cells in clinical response.

As several bispecific antibodies have been developed in MM therapy, understanding the mechanisms of resistance and sensitivity has become increasingly important, and it is anticipated that their use in appropriate combinations with SoC drugs would be beneficial. Consistent with previous studies, this study showed that IMiDs are compatible as combination therapy^48^. IMiDs are particularly effective in promoting immune activation, which is expected to lead to a durable response and suppression of relapse.

In conclusion, our newly produced humanized monoclonal bispecific antibody, BsAb5003, demonstrated sufficient potency against MM cell lines and primary MM as a T cell engager. BsAb5003 differs from Talquetamab in several aspects. Firstly, BsAb5003 was constructed in an asymmetric format, "scFv-Fab-Fc," with improved effectorlessness by triple mutations of Leu234Ala, Leu235Ala, and Asp265Ser in IgG1^23,44^. On the other hand, Talquetamab is in a symmetric format, "Fab-Fab-Fc," with effectorless mutations of Leu234Ala and Leu235Ala in IgG4^49^. Furthermore, the GPRC5D and CD3 binders used in BsAb5003 are different from those used in Talquetamab. Importantly, our GPRC5D binder targets the N-terminal region of GPRC5D and demonstrated strong target binding signal in FCM. In contrast, referring to the published information^49^, the GPRC5D binder GCDB72 for Talquetamab showed the weakest target binding affinity among their binders with a range of binding affinities in FCM. These differences, along with our results, highlight the uniqueness and potential efficacy and safety of BsAb5003. The most important factor for the efficacy of this antibody is expression of the tumor-associated antigen GPRC5D. Meanwhile, immune status is also likely to influence the level of activation. Overall, in this study, we demonstrated the therapeutic potential of GPRC5D targeted T cell-redirecting antibody as a monotherapy and combination therapy for MM in preclinical studies.

## Materials and methods

### Antibodies and reagents

BsAb5003 (humanized anti-GPRC5D × CD3 bispecific antibody) was developed based on the heterodimeric Fc variant via the Azymetric™ technology^20^. Anti-GPRC5D and anti-CD3 antibodies were derived from rat immunization and humanized BsAb5003 constructs consist of three chains: an anti-GPRC5D antibody heavy chain, an anti-GPRC5D light chain, and an anti-CD3 scFv-Fc fusion chain. The anti-GPRC5D arm is in Fab format, and the anti-CD3 is in scFv format, in which VH connects with VL using a 15-amino-acid linker (G_4_S)_3_. BsAb5003 constant region is the IgG1 subtype with EFECT™ mutation, which abolishes Fc-mediated effector functions^23^. BsAb5003 and anti-human GPRC5D monoclonal antibodies with mouse Fc (Clone 2B1) for flow cytometry (FCM) or rabbit Fc (Clone 8C2) for immunohistochemistry (IHC) were produced by Daiichi Sankyo (Tokyo, Japan), via mammalian cell-based expression systems. Ultra-LEAF Purified Human IgG1 Isotype Control Recombinant Antibody (BioLegend, San Diego, California, USA) and mouse IgG1 kappa Isotype Control (Invitrogen, Carlsbad, California, USA) were used as isotype control of BsAb5003 and mouse anti-human GPRC5D antibody, respectively. Lenalidomide (Santa Cruz Biotechnology, Dallas, Texas, USA), pomalidomide (Tokyo Chemical Industry, Tokyo, Japan), bortezomib (Selleck Chemicals, Houston, Texas, USA) and carfilzomib (LC Laboratories, Woburn, Massachusetts, USA) were purchased and dissolved in dimethyl sulfoxide (DMSO).

### Cell lines

KMS-11, KMS-21BM, KMS-26, and KMS-28BM were obtained from JCRB Cell Bank, NCI-H929 and U266B1 were from ATCC (Manassas, Virginia, USA); and OPM2 and LP-1 were from DSMZ (Braunschweig, Germany). Cells were cultured as described in the supplier’s instructions.

### Patients and specimens

Bone marrow (BM) aspirates from 49 MM patients were collected at Nagoya City University (Aichi, Japan) after the provision of informed consent in accordance with the Declaration of Helsinki, and all studies on human subjects were conducted in accordance with the protocol approved by the Institutional Ethical Committee of Nagoya City University, and Daiichi Sankyo. The clinical characteristics of patients enrolled in this study are shown in Table 1. Bone marrow mononuclear cells (BMMNCs) were collected from fresh buffy coat of each BM aspirates.

### In vitro MM cytotoxicity and T cell activation assay with MM cell lines

Cryopreserved human peripheral blood mononuclear cells (hPBMCs) from a healthy donor were purchased from Cellular Technology (Cleveland, Ohio, USA). MM cell lines labeled with PKH67 Green Fluorescent Cell Linker Kit (Sigma-Aldrich, Burlington, Massachusetts, USA) were adjusted to 4 × 10^5^ cells/mL and hPBMCs were adjusted to 1 × 10^6^ cells/mL with culture medium. A total of 100 μL of hPBMCs (1 × 10^5^ cells/well) and 50 μL of PKH67-labeled target cells (2 × 10^4^ cells/well) were seeded into 96-well round-bottomed microplates (effector cells:target cells = 5:1) with or without BsAb5003. To evaluate the effects of combination treatment with IMiDs or PI, the prepared diluents of test compounds (final concentration of DMSO: 0.1%) were added to appropriate wells. Culture plates were centrifuged at 300 g for 3 min and cells were co-cultured.

On day 1, a small portion (50 μL) of each culture supernatant was collected from each well for cytokine multiplex assay. After 3 days of co-culture, cell pellets were stained with LIVE/DEAD and labeled antibodies for the detection of CD8, CD4, and CD25. Samples were analyzed on BD FACS Canto II using FlowJo 10.7.1 software (BD Life Sciences, Ashland, Oregon, USA, https://www.flowjo.com/).

Cytotoxicity was determined as the percent of remaining PKH67-labeled MM cells compared with that in the untreated control, which was calculated using the following formula: % Cytotoxicity = 100 − (viable target cells of treatment sample × 100/viable target cells of untreated control).

The activation levels of CD8 + T and CD4 + T cells were determined by the expression level of CD25, which was detected as the mean fluorescence intensity (MFI).

### Ex vivo assays with patient samples

Patient-derived BMMNCs were prepared at 1 × 10^6^ cells/mL in RPMI Medium 1640 (Thermo Fisher, Waltham, Massachusetts, USA) supplemented with 20% (v/v) FBS (HyClone, Logan, Utah, USA) and 1% Penicillin–Streptomycin Solution (FUJIFILM Wako, Osaka, Japan). A total of 100 μL of BMMNC suspension was seeded into 96-well round-bottomed microplates with 100 μL of BsAb5003 antibody solutions or culture medium as an untreated control. The 96-well plates were centrifuged at 300 g for 3 min and cultured for 2–4 days. Supernatants were gently collected from all wells for cytokine multiplex assay. Cell pellets were subjected to LIVE/DEAD staining and divided into two. In one portion, cells were stained with labeled antibodies for CD138, SLAMF7, and CD11b to evaluate the cytotoxicity of MM cells. In the other portion, cells were stained with labeled antibodies for CD4, CD8, CD25, and CD11b to evaluate the activation of T cell subsets.

MM cell cytotoxicity was calculated as follows: % MM cell cytotoxicity = 100 − (viable CD138 + cells of treatment sample × 100/viable CD138 + cells of untreated control). If the value of MM cell cytotoxicity became negative, the cytotoxicity was defined as no effect (0%).

T cell activation was determined by the percentage of CD25 + cells among CD8 + T cells and CD4 + T cells. The threshold of CD25 expression was set as < 0.5% in isotype control samples.

### RNA sequencing of patient-derived BMMNCs

CD138 + cells in BMMNCs were enriched by the CD138 MicroBeads (Miltenyi Biotec, Bergisch Gladbach, Germany), in accordance with the manufacturer’s instructions. Total RNA of both BMMNCs and CD138 + cells was extracted using AllPrep DNA/RNA Micro Kit (QIAGEN, Hilden, Germany) or AllPrep DNA/RNA Mini Kit (QIAGEN), in accordance with the manufacturer’s instructions.

A library for RNA sequencing was prepared using SMART-seq v4 (Takara Bio USA, San Jose, California, USA) and paired-end sequencing of RNA reads of 150 bases was performed using a NovaSeq 6000 sequencer (Illumina, San Diego, California, USA). Estimated counts and TPM values were obtained by mapping the reads to the GRCh38 reference genome using the DRAGEN Bio-IT Platform (Illumina, https://www.illumina.com/products/by-type/informatics-products/dragen-secondary-analysis.html). To identify gene expression associated with drug efficacy, cytotoxicity at 10 nM treatment by BsAb5003 was selected as value of the efficacy, and linear regression analysis was performed with cytotoxicity at 10 nM treatment by BsAb5003 as the explanatory variable using the limma package (version 3.46.0, https://bioconductor.org/packages/release/bioc/html/limma.html) in Bioconductor (version 3.12, https://www.bioconductor.org/) in R (version 4.0.5, https://www.r-project.org/). Sample condition (fresh or frozen) was added to the covariates to remove bias due to the sample condition.

### KMS-26 xenograft model

Female NSG (NOD.Cg-Prkdc^scid^ Il2rg^tm1Wjl^/SzJ) mice were purchased from The Jackson Laboratory Japan, Inc., and used at 6 to 7 weeks of age. All animal experimental procedures in this study were approved by the Institutional Animal Care and Use Committee at Daiichi Sankyo Co., Ltd. (approval number: 20000399) and were performed in accordance with the guidelines established by the Institutional Animal Care and Use Committee of Daiichi Sankyo Co., Ltd., which are in compliance with the ARRIVE guidelines. A total of 3 × 10^6^ KMS-26 cells suspended in 50% Matrigel basement membrane matrix (Corning, New York, USA) were inoculated subcutaneously (SC) into the right flank of NSG mice. On day 9, mice were randomized to groups by tumor size at similar starting tumor volumes of ~ 100 mm^3^ (n = 6/group). On day 10, each mouse received intravenous (IV) injection of 1 × 10^7^ human T cells prepared from hPBMCs using Dynabeads Human T-Activator CD3/CD28 (Thermo Fisher), followed by SC administration of PBS or BsAb5003 at 0.003 mg/kg, 0.01 mg/kg, 0.03 mg/kg, or 0.1 mg/kg. Each mouse received SC administration into the back once every week for two cycles (on Days 10 and 17). Tumor length and width and body weight were measured. The estimated tumor volume for each mouse was calculated using the following formula: tumor volume (mm^3^) = tumor length × tumor width^2^ × 1/2. Tumor growth inhibition (TGI) in each group was calculated as TGI (%) = (1 − T/C) × 100, where T is mean estimated tumor volume of the BsAb5003-treated group and C is mean estimated tumor volume of the control group.

### Statistical analysis

Statistical analyses were performed in SAS System Release 9.2 (SAS Institute, Cary, North Carolina, USA, https://www.sas.com/). Histograms and dot plots were created using FlowJo 10.7.1 software and other graphs were drawn using GraphPad PRISM (version 9.1.0, GraphPad Software, Boston, Massachusetts, USA, https://www.graphpad.com/). The maximal efficacy (E_max_), half maximal effective concentration (EC_50_), and 95% confidence interval (CI) values on each assay were estimated by Sigmoid E_max_ model. Williams test was conducted to evaluate the statistical significance of BsAb5003 treatment compared to non-treated control in in vitro study. Raw probability values (*P-*values) of less than 0.025 were considered statistically significant. In the combination in vitro assay, Dunnett’s multiple comparison test was performed to determine the significance of the effects of lenalidomide, pomalidomide, bortezomib or carfilzomib under the conditions in which BSAb5003 was treated. Raw *P-*values of less than 0.05 were considered statistically significant. The combined effects of BsAb5003 with lenalidomide, pomalidomide, bortezomib or carfilzomib was determined by Bliss independence model, as described before^50^. In in vivo study, the correlations between two parameters were confirmed by Spearman’s rank correlation test and Dunnett’s multiple comparison test was used to confirm the dose–response of BsAb5003. Raw *P-*values of less than 0.05 were considered statistically significant.

### Supplementary Information

Supplementary Information.

## Data Availability

The datasets used and analyzed during this study are available from the corresponding author upon reasonable request.

## References

[1] Vo JN (2022). The genetic heterogeneity and drug resistance mechanisms of relapsed refractory multiple myeloma. Nat. Commun..

[2] Rajkumar SV (2020). Multiple myeloma: 2020 update on diagnosis, risk-stratification and management. Am. J. Hematol..

[3] Sonneveld P (2016). Treatment of multiple myeloma with high-risk cytogenetics: A consensus of the International Myeloma Working Group. Blood.

[4] Bhatt P, Kloock C, Comenzo R (2023). Relapsed/refractory multiple myeloma: A review of available therapies and clinical scenarios encountered in myeloma relapse. Curr. Oncol..

[5] Atamaniuk J (2012). Overexpression of G protein−coupled receptor 5D in the bone marrow is associated with poor prognosis in patients with multiple myeloma. Eur. J. Clin. Investig..

[6] Cohen Y, Gutwein O, Garach-Jehoshua O, Bar-Haim A, Kornberg A (2013). GPRC5D is a promising marker for monitoring the tumor load and to target multiple myeloma cells. Hematology.

[7] Smith EL (2018). CAR T cell therapy targeting G protein-coupled receptor class C group 5 member D (GPRC5D), a novel target for the immunotherapy of multiple myeloma. Blood.

[8] Mailankody S (2022). GPRC5D-targeted CAR T cells for myeloma. N. Engl. J. Med..

[9] Inoue S, Nambu T, Shimomura T (2004). The RAIG family member, GPRC5D, is associated with hard-keratinized structures. J. Invest. Dermatol..

[10] Kodama T (2019). Anti-GPRC5D/CD3 bispecific T-cell-redirecting antibody for the treatment of multiple myeloma. Mol. Cancer Ther..

[11] Pillarisetti K (2020). A T-cell–redirecting bispecific G-protein–coupled receptor class 5 member D x CD3 antibody to treat multiple myeloma. Blood.

[12] Smith EL (2019). GPRC5D is a target for the immunotherapy of multiple myeloma with rationally designed CAR T cells. Sci. Transl. Med..

[13] Keam SJ (2023). Talquetamab: First approval. Drugs.

[14] Mullard A (2023). GPRC5D-targeted bispecific bolsters T-cell-engager pipeline. Nat. Rev. Drug Discov..

[15] Zhang M (2023). GPRC5D CAR T cells (OriCAR-017) in patients with relapsed or refractory multiple myeloma (POLARIS): A first-in-human, single-centre, single-arm, phase 1 trial. Lancet Haematol..

[16] Lee H (2023). Mechanisms of antigen escape from BCMA- or GPRC5D-targeted immunotherapies in multiple myeloma. Nat. Med..

[17] Cho SF, Yeh TJ, Anderson KC, Tai YT (2022). Bispecific antibodies in multiple myeloma treatment: A journey in progress. Front. Oncol..

[18] Chari A (2023). Plain language summary of the MonumenTAL-1 study of talquetamab in people with relapsed or refractory multiple myeloma. Future Oncol..

[19] Vafa O, Trinklein D (2020). Perspective: Designing T-cell engagers with better therapeutic windows. Front. Oncol..

[20] Long M, Mims AS, Li Z (2022). Factors affecting the cancer immunotherapeutic efficacy of T cell bispecific antibodies and strategies for improvement. Immunol. Invest..

[21] Ellerman D (2019). Bispecific T-cell engagers: Towards understanding variables influencing the in vitro potency and tumor selectivity and their modulation to enhance their efficacy and safety. Methods.

[22] Kreudenstein TSV (2013). Improving biophysical properties of a bispecific antibody scaffold to aid developability: Quality by molecular design. MAbs.

[23] Ng, G. Y. K. *et al.* Bispecific Asymmetric Heterodimers Comprising Anti-CD3 Constructs. *U. S. Pat.* US20190248897 (2019).

[24] Rajikumar SV, Kumar S (2020). Multiple myeloma current treatment algorithms. Blood Cancer J..

[25] Tsherniak A (2017). Defining a cancer dependency map. Cell.

[26] Foureau DM (2020). Ex vivo efficacy of BCMA-bispecific antibody TNB-383B in relapsed/refractory multiple myeloma. eJHaem.

[27] Frerichs KA (2020). Preclinical activity of JNJ-7957, a novel BCMA×CD3 bispecific antibody for the treatment of multiple myeloma, is potentiated by daratumumab. Clin. Cancer Res..

[28] Yamamoto J (2020). ARID2 is a pomalidomide-dependent CRL4CRBN substrate in multiple myeloma cells. Nat. Chem. Biol..

[29] Quach H (2010). Mechanism of action of immunomodulatory drugs (IMiDS) in multiple myeloma. Leukemia.

[30] Cho SF (2020). The immunomodulatory drugs lenalidomide and pomalidomide enhance the potency of AMG 701 in multiple myeloma preclinical models. Blood Adv..

[31] Dimopoulos K (2018). Dual inhibition of DNMTs and EZH2 can overcome both intrinsic and acquired resistance of myeloma cells to IMiDs in a cereblon-independent manner. Mol. Oncol..

[32] Zhu YX (2011). Cereblon expression is required for the antimyeloma activity of lenalidomide and pomalidomide. Blood.

[33] Field-Smith A, Morgan GJ, Davies FE (2006). Bortezomib (Velcade™) in the treatment of multiple myeloma. Ther. Clin. Risk Manag..

[34] Perel G, Bliss J, Thomas CM (2016). Carfilzomib (Kyprolis): A novel proteasome inhibitor for relapsed and/or refractory multiple myeloma. P T.

[35] Gandolfi S, Laubach JP, Hideshima T, Chauhan D, Anderson KC, Richardson PG (2017). The proteasome and proteasome inhibitors in multiple myeloma. Cancer Metastasis Rev..

[36] Hipp S (2017). A novel BCMA/CD3 bispecific T-cell engager for the treatment of multiple myeloma induces selective lysis in vitro and in vivo. Leukemia.

[37] Feldmann A, Arndt C, Töpfer K, Stamova S, Krone F, Cartellieri M (2012). Novel humanized and highly efficient bispecific antibodies mediate killing of prostate stem cell antigen-expressing tumor cells by CD8+ and CD4+ T cells. J. Immunol..

[38] Martens AWJ (2022). Redirecting T-cell activity with anti-BCMA/Anti-CD3 bispecific antibodies in chronic lymphocytic leukemia and other B-cell lymphomas. Cancer Res. Commun..

[39] Panowski SH (2019). Preclinical efficacy and safety comparison of CD3 bispecific and ADC modalities targeting BCMA for the treatment of multiple myeloma. Mol. Cancer Ther..

[40] Bacac M (2016). A novel carcinoembryonic antigen T-cell bispecific antibody (CEA TCB) for the treatment of solid tumors. Clin. Cancer Res..

[41] Schaller TH, Snyder DJ, Spasojevic I, Gedeon PC, Sanchez-Perez L, Sampson JH (2020). First in human dose calculation of a single-chain bispecific antibody targeting glioma using the MABEL approach. J. Immunother. Cancer.

[42] Leclercq G (2022). Dissecting the mechanism of cytokine release induced by T-cell engagers highlights the contribution of neutrophils. Oncoimmunology.

[43] Ng, G. Y. K. *et al.* Bi-Specific CD3 and CD19 Antigen-Binding Constructs. *Int. Pat.* WO/2015/109131 (2015).

[44] Edavettal S (2022). Enhanced delivery of antibodies across the blood-brain barrier via TEMs with inherent receptor-mediated phagocytosis. Med.

[45] Schüler J, Ewerth D, Waldschmidt J, Wäsch R, Engelhardt M (2013). Preclinical models of multiple myeloma: A critical appraisal. Expert Opin. Biol. Ther..

[46] Durie BGM (2006). International uniform response criteria for multiple myeloma. Leukemia.

[47] Aran D, Hu Z, Butte AJ (2017). xCell: Digitally portraying the tissue cellular heterogeneity landscape. Genome Biol..

[48] Verkleij CPM (2021). Preclinical activity and determinants of response of the GPRC5DxCD3 bispecific antibody talquetamab in multiple myeloma. Blood Adv..

[49] Attar, R. *et al*. Anti-GPRC5D Antibodies, Bispecific Antigen Binding Molecules that Bind GPRC5D and CD3, and Uses Thereof. *Int. Pat.* WO/2018/017786 (2018).

[50] Foucquier J, Guedj M (2015). Analysis of drug combinations: Current methodological landscape. Pharmacol. Res. Perspect..

