# Supplementary material for: A novel T cell-redirecting anti-GPRC5D × CD3 bispecific antibody with potent antitumor activity in multiple myeloma preclinical models
Source: Sci Rep. 2024 Mar 1;14:5135. doi: 10.1038/s41598-024-55143-0 (PMC10907593; doi:10.1038/s41598-024-55143-0)
Supplement: Supplementary file 1 — Supplementary Information. [file 41598_2024_55143_MOESM1_ESM.docx]

## Supplemental methods

### Reagents

Lenalidomide (Santa Cruz), pomalidomide (Tokyo Chemical Industry), bortezomib (Selleck Chemicals), and carfilzomib (LC Laboratories) were purchased and dissolved in dimethyl sulfoxide (DMSO) (FUJIFILM Wako). Ultra-LEAF Purified Human IgG1 Isotype Control Recombinant Antibody (BioLegend) and mouse IgG1 Kappa Isotype Control (Invitrogen) were used as isotype control of BsAb5003 and anti-human GPRC5D antibody, respectively.

### ddPCR

Total RNA was extracted using RNeasy Mini kit (QIAGEN) and cDNA was synthesized using SuperScript IV VILO Master Mix (Thermo Fisher), in accordance with the manufacturer’s protocol. Multiplex assay was performed by reaction with cDNA sample, GPRC5D (Hs03045338_m1, Thermo Fisher), HPRT1 (dHsaCPE5192872, Bio-Rad), TBP (dHsaCPE80933618, Bio-Rad), and GUSB (dHsaCPE44078889, Bio-Rad) detection probe sets in the same well. Data were analyzed using QX ONE software 1.10 Standard Edition (Bio-Rad). The gene expression levels were normalized to that of the geometric mean across the three housekeeping control transcripts (HPRT, TBP, and GUSB); then, the relative expression of each gene was calculated as percent control compared with the value of U266B1

### Immunohistochemistry

IHC (CD3 and GPRC5D) staining was carried out using Leica BOND Rx staining platform (Leica Microsystems K.K.). Slides of FFPE samples were incubated with the anti-GPRC5D monoclonal antibody with rabbit Fc (Clone 8C2, Daiichi Sankyo) and Bond Primary Antibody Diluent (Leica) or with CD3 (LN10) BOND Ready-to-Use Primary Antibody (Leica), and then detected with BOND Polymer Refine Detection Kit (Leica). The stained section was digitized with a digital slide scanner, NanoZoomer-XR (C12000-03, Hamamatsu Photonics K.K.), and image analysis was performed with it. Membranous GPRC5D staining intensity in MM tumor cell lines was scored with the Membrane IHC module in HALO image analysis software (Indica Labs). The staining intensity of each tumor cell was classified as negative or positive at three intensity levels (strong, moderate, or weak). Based on the percentage of tumor cells at each staining intensity level, an H score was calculated using the following formula:

H score = 3 × (Percentage of strongly positive tumor cells) + 2 × (Percentage of moderately positive tumor cells) + 1 × (Percentage of weakly positive tumor cells).

CD3-positive cells were manually scored as negative if there were fewer than 10 CD3-positive cel1s in the tumor area on average, or positive if there were 10 or more CD3-positive cel1s in the tumor area on average, by optical observation in a 100× field of view with a microscope.

### Cell surface protein expression analysis using flow cytometry

Dead cells were excluded by staining with LIVE/DEAD Fixable Near-IR Dead Cell Stain Kit (Thermo Fisher). hPBMCs were stained with BV421 Anti-human CD8 Antibody (clone: SK1, BioLegend), PerCP/Cy5.5 Anti-human CD4 Antibody (clone: SK3, BioLegend) and APC Anti-human CD19 Antibody (clone: J3-119, BioLegend). PE Anti-human IgG Fc (BioLegend) was used as a secondary antibody to detect the binding of BsAb5003 on MM cell lines or hPBMCs.

To quantify GPRC5D expression quantification on MM cell lines or patient derived BM cells, anti-human GPRC5D monoclonal antibody with mouse Fc (Clone 2B1, Daiichi Sankyo) with QIFIKIT (Dako) was used, in accordance with the manufacturer’s protocol.

Patient-derived BM cells were also stained with MM surface marker antibody cocktails containing V450 anti-human CD138 (Clone MI15, BD Bioscience), PE anti-human SLAMF7 (Clone 162, Abcam plc.), PE/Cy7 anti-human CD38 (Clone LS198-4-3, Beckman Coulter), APC anti-human BCMA (Clone 19F2, BioLegend.) and PerCP/Cy5.5 anti-human CD11b (Clone ICRF44, BioLegend) or with T cell subset antibody cocktails containing APC anti-human CD3 antibody (Clone OKT3, BioLegend), PerCP/Cy5.5 anti-human CD4 antibody (Clone SK3, BioLegend), BV421 anti-human CD8 antibody (Clone SK1, BioLegend) and BV650 anti-human CD25 antibody (CloneM-A251, BD Bioscience). Samples were analyzed on flow cytometer BD LSR Fortessa X-20 or BD FACS Canto II (Becton Dickinson) using FlowJo 10.7.1 software (FlowJo). The gating strategy is shown in Supplemental Fig. 8.

### Cytokine measurement

The concentrations of Interferon γ (IFNγ), IL-2 and Tumor Necrosis Factor α (TNFα) in culture supernatants were assayed using MILLIPLEX MAG Human Cytokine/Chemokine panel kit (Merck), as per the vendor’s instructions. Samples were evaluated using the Luminex 200 system (Merck) and analyzed with Luminex xPONENT software (version 3.1, Merck).

### xCELL analysis

xCELL analysis was performed to estimate the immune cell profile of the PBMC samples using xCELL R package (version 1.1.0, https://github.com/dviraran/xCell).

## Supplemental Figures

**Supplemental Fig. 1: Binding activities of BsAb5003 to N-terminal peptides of human GPRC5D and GPRC5A were evaluated by ELISA.**

**a** Amino acid sequences of human GPRC5D and GPRC5A N-terminal peptides. The peptides are disulfide-linked and biotinylated. An asterisk (*) indicates a disulfide link.

**b** N-terminal peptide of human GPRC5D or GPRC5A was coated on a plate, followed by treatment with 0.8, 4, 20, and 100 ng/mL BsAb5003. Data represent the mean ± SD of triplicate values of absorption at 405 nm.

**Supplemental Fig. 2: mRNA expression level and IHC staining of GPRC5D in several MM cell lines.**

**a** GPRC5D mRNA expression levels in eight MM cell lines (MM.1R, NCI-H929, KMS-26, KMS-28BM, KMS-11, LP-1, U266B1, and KMS-11) were measured by ddPCR. The GPRC5D expression levels were normalized to that of the geometric mean across the three housekeeping control transcripts (HPRT, TBP, and GUSB), and relative GPRC5D expression is shown as a percent control compared with the value of U266B1. Samples were prepared in quadruplicate and the data represent the mean ± standard deviation. **b** Representative GPRC5D IHC staining of each MM cell line. (A) NCI-H929, (B) KMS-26, (C) KMS-28BM, (D) KMS-21BM, (E) LP-1, (F) U266B1, and (G) KMS-11.


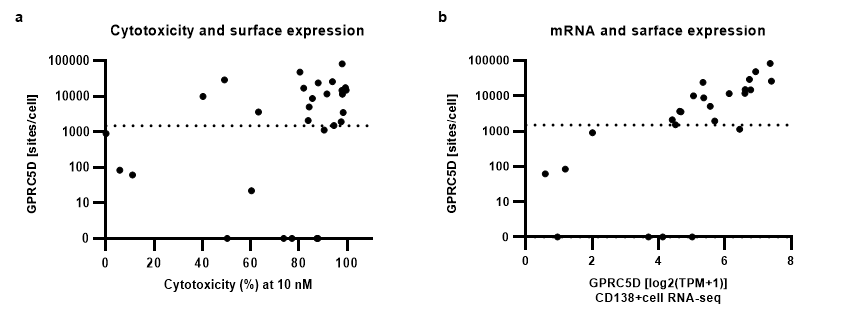


**Supplemental Fig. 3:** **Relationship between QIFIKIT-determined cell surface GPRC5D expression level and cytotoxicity induced by BsAb5003 or GPRC5D RNA expression level on BMMNCs.**

Cell surface GPRC5D expression on BMMNCs derived from 29 MM patients was determined using QIFIKIT. Dotted line is the lower limit of detection (1500 sites/cell). When the calculated value was negative, cell surface GPRC5D expression level was defined as 0 sites/cell. RNA sequence data of isolated CD138+ cells was obtained from 26 samples out of these 29 samples. a Correlation of cytotoxicity induced by 10 nM BsAb5003 treatment and QIFIKIT-determined cell surface GPRC5D expression level. b Correlation of RNA expression level and cell surface expression level of GPRC5D.

**
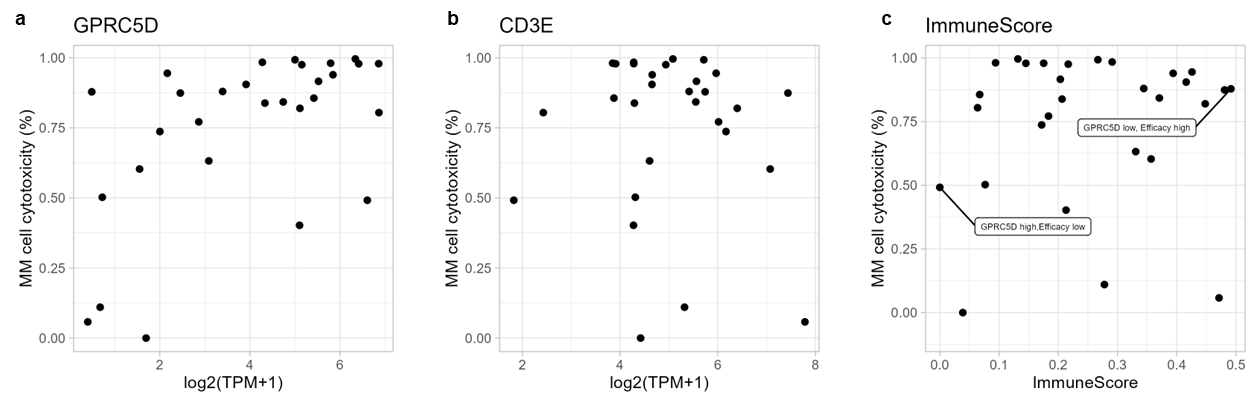
**

**Supplemental Fig. 4:** **Correlation between BsAb5003 efficacy and expression of GPRC5D, CD3, and xCELL parameter.**

Association between **a** GPRC5D and **b** CD3e mRNA expression and MM cell cytotoxicity (%). **c** Association between ImmuneScore calculated from xCELL analysis and MM cell cytotoxicity (%). The sample with the lowest GPRC5D among those with more than 50% of MM cell cytotoxicity was labeled as “GPRC5D low, Efficacy high”, and the sample with the highest GPRC5D among those with less than 50% of MM cell cytotoxicity was labeled as “GPRC5D high, Efficacy low”.


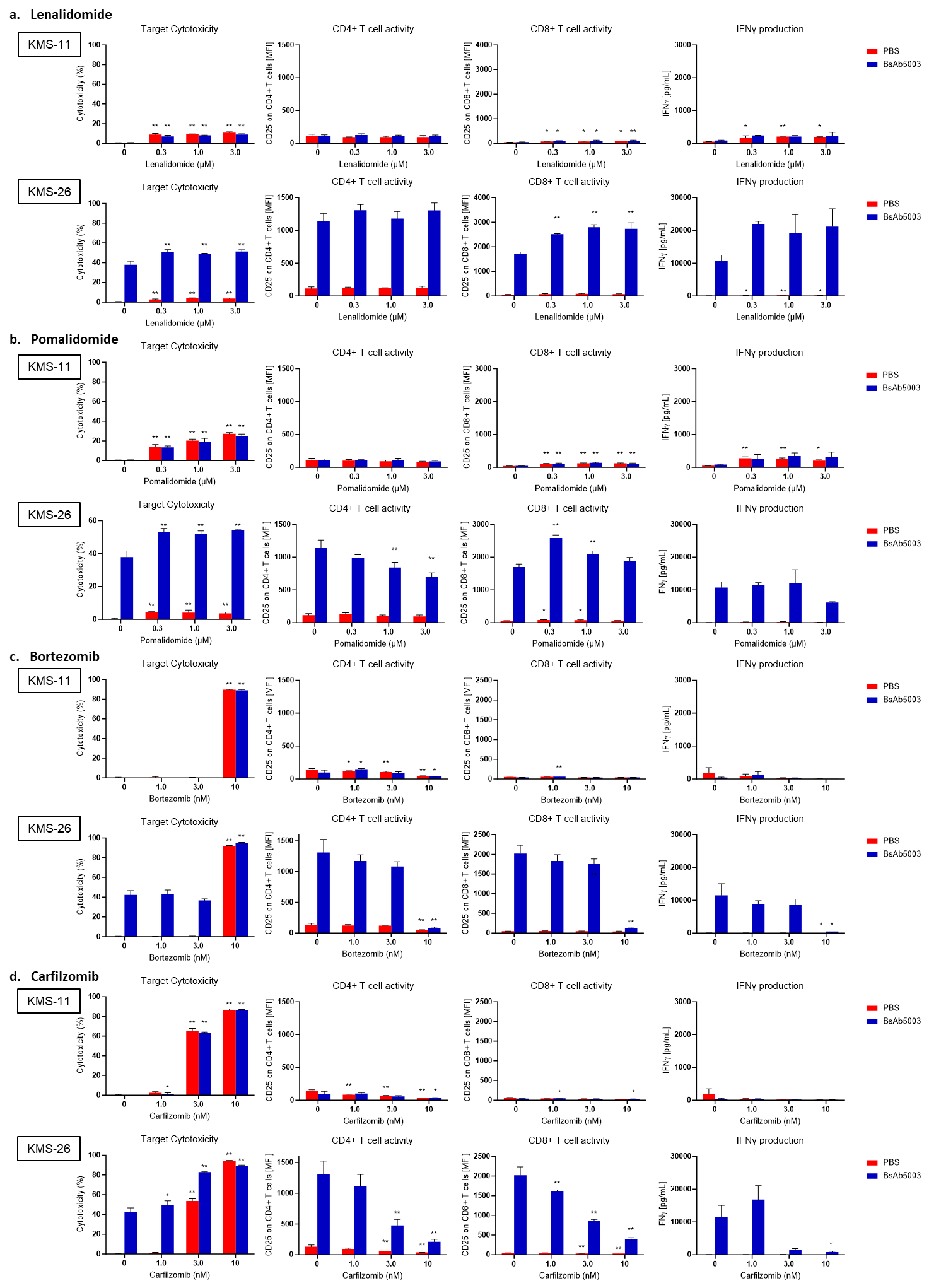


**Supplemental Fig. 5: Effect of BsAb5003 combined with several MM treatment drugs in KMS-11 cells and KMS-26 cells.**

GPRC5D-negative KMS-11 cells and GPRC5D positive KMS-26 cells were co-cultured with hPBMCs with (blue bar) or without (red bar) 5 pM BsAb5003 in combination with **a** lenalidomide, **b** pomalidomide, **c** bortezomib, or **d** carfilzomib. On the first day of incubation, culture supernatants were collected for cytokine measurement. After 3 days of incubation, cells were collected for cytotoxicity and CD4+ T cell activation measurement. The assay was conducted in triplicate and the data represent the mean ± standard error. Statistical significance between the group without combination drug and the groups with combination drug was assessed using Parametric Dunnett's test for each condition treated with PBS and BsAb5003 (*: P < 0.05, **: P < 0.001).


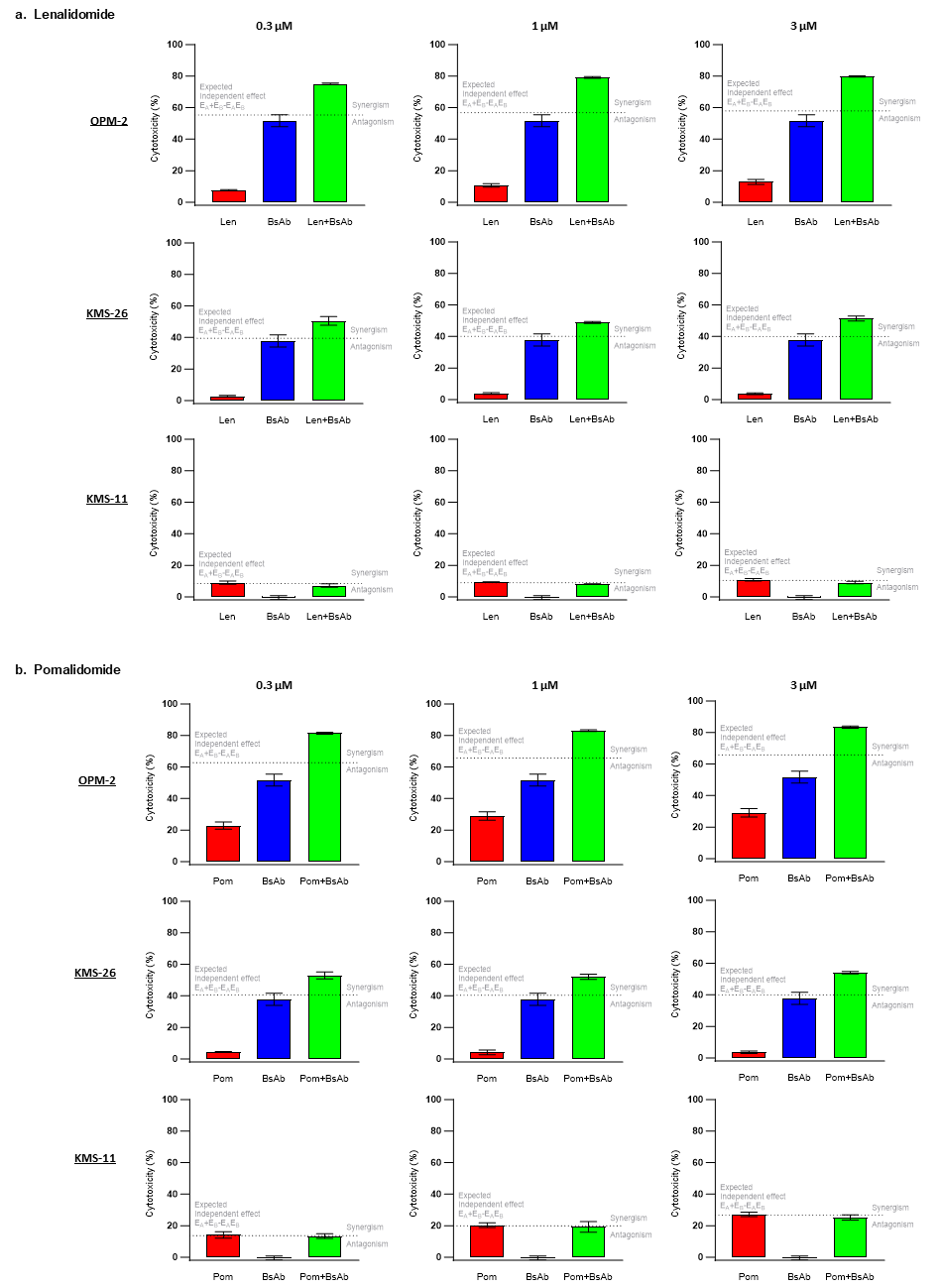


**Supplemental Fig. 6: Combination index (CI) calculated by Bliss independence model.**


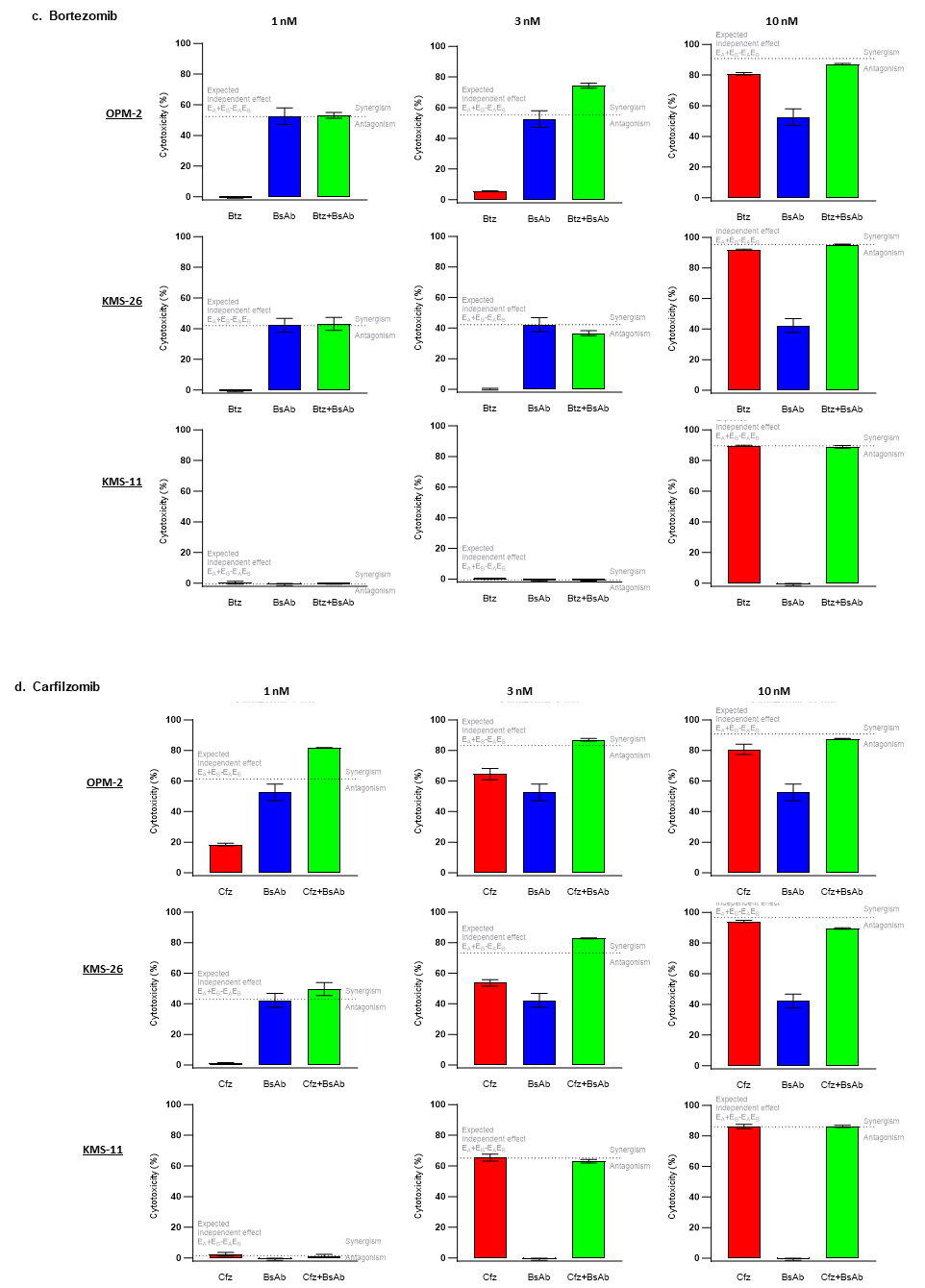


**(Cont.) Supplemental Fig. 6: Combination index (CI) calculated by Bliss independence model.**

Estimated Independent effect ($E_{A}+E_{B}-E_{A}E_{B}$) of BsAb5003 and **a** lenalidomide, **b** pomalidomide, **c** bortezomib, or **d** carfilzomib was calculated according to Bliss independence model and the combination effect was determined as following:

$E_{A}+E_{B}-E_{A}E_{B}<E_{AB}$　…… Synergism

$E_{A}+E_{B}-E_{A}E_{B}>E_{AB}$　……Antagonism

E_A_: The observed cytotoxicity of lenalidomide, pomalidomide, bortezomib or carfilzomib at indicated concentration without BsAb5003 (Red bar, mean ± standard deviation of triplicates).

E_B_: The observed cytotoxicity of 5 pM BsAb5003 (Blue bar, mean ± standard deviation of triplicates).

E_AB_: The observed cytotoxicity of Lenalidomide, Pomalidomide, Bortezomib or Carfilzomib at indicated concentration with 5 pM BsAb5003 (Green bar, mean ± standard deviation of triplicates). Estimated Independent effect ($E_{A}+E_{B}-E_{A}E_{B}$) is indicated as dotted line in each graph.


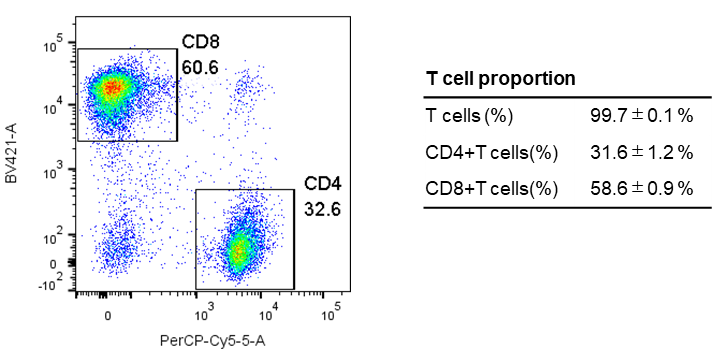


**Supplement Fig. 7: Profile of human T cells prepared for the transplantation**

Human T cells for the transplantation into the xenograft model were prepared by stimulating hPBMC with Dynabeads Human T-Activator CD3/CD28 and IL-2 for 13 days. Left panel is the representative dot plot of CD4+ and CD8+ T cells in expanded hPBMCs. Right table is the list of mean ± standard deviation of triplicate data.


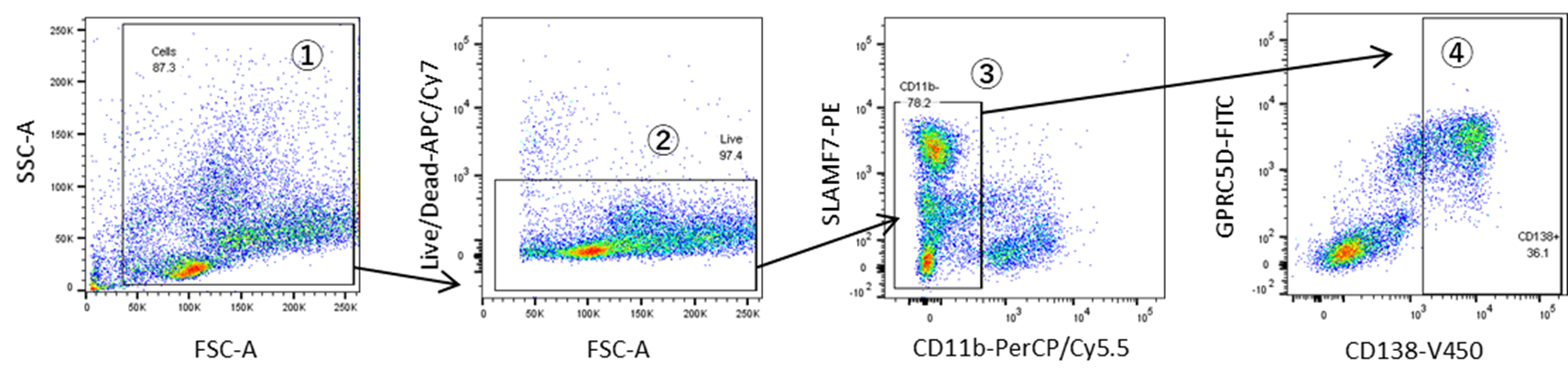


**Supplement Fig. 8: Gating strategies for malignant plasma cells**

Cell surface marker expression levels on malignant plasma cells were detected by a flow cytometer using FlowJo 10.7.1 software according to the gating strategy shown in this figure. First, (1) cells were gated (FSC-A vs. SSC-A plot) and then (2) live cells (APC/Cy7^−^) were gated. Next, (3) CD11b-negative cells (PerCP/Cy5.5^−^) were gated to exclude myeloid cells. Finally, (4) malignant plasma cells were determined as the CD138+ (BV450+) cell population. Expression levels of GPRC5D, BCMA, CD38, and SLAMF7 on malignant plasma cells were determined as MFI of FITC, APC, PE/Cy7, and PE, respectively. SSC-A, area of side scatter; FSC-A, area of forward scatter; MFI, mean fluorescence intensity.
